# Effectiveness of Psychosocial Interventions in Preventing Postpartum Depression Among Teenage Mothers—Systematic Review and Meta-analysis of Randomized Controlled Trials

**DOI:** 10.1007/s11121-024-01728-0

**Published:** 2024-10-03

**Authors:** Lebeza Alemu Tenaw, Fei Wan Ngai, Chan Bessie

**Affiliations:** 1 School of Nursing, The Hong Kong Polytechnic University, Kowloon, Hong Kong; 2 School of Public Health, College of Health Sciences, Woldia University, Woldia, Ethiopia

**Keywords:** Teenage pregnancy, Postpartum depression, Systematic review, Meta-analysis

## Abstract

Postpartum depression is a significant public health issue that occurs within the first 12 weeks after childbirth. It is more prevalent among teenage mothers compared to adults. However, the findings of the existing interventional studies cannot be readily applied to teenage mothers due to their unique psychosocial concerns. Furthermore, these findings have shown inconsistencies regarding the benefit of psychological and psychosocial interventions in reducing the incidence of postpartum depression. The current review is aimed at investigating the effectiveness of psychosocial interventions in preventing postpartum depression, specifically among teenage mothers. The preferred reporting items for systematic reviews and meta-analysis manuals were utilized to identify and select relevant articles for this review. The articles were retrieved using population, intervention, control, and outcome models. The quality of each article was assessed using the Cochrane risk of bias tool. Statistical analysis was conducted using STATA version 17. The effect size of the intervention was estimated using the standard mean difference in depression scores between the intervention and control groups. Heterogeneity among the studies was assessed using the *I*^2^ statistic and *Q* statistic, while publication bias was evaluated through funnel plot asymmetry and Egger’s test. A total of nine eligible articles were included. While psychosocial interventions have been demonstrated to decrease the incidence of postpartum depression compared to usual maternal health care, it is worth noting that the mean difference in depression scores was significant in only three of the included studies. The meta-analysis revealed that psychosocial interventions were effective at preventing postpartum depression, with a pooled effect size of − 0.5 (95% CI: − 0.95, − 0.06) during the final postpartum depression assessment. The heterogeneity was substantial, with an *I*^2^ value of 82.3%. Although publication bias was not observed, small studies had a significant effect on the pooled effect size. The findings of this review suggest that psychosocial interventions can effectively prevent PPD, particularly within the first 3 months of the postpartum period. This review highlights the scarcity of interventional studies in low-income countries, indicating the need for further research in diverse communities.

**Supplementary Information:**

The online version contains supplementary material available at 10.1007/s11121-024-01728-0.

## Introduction

Teenage pregnancy, occurring between the ages of 13 and 19 years, continues to be a significant public health issue, with approximately 16 million adolescent births reported annually worldwide (Akella & Jordan, 2015; Riley, 2020). Teenage women are often not well equipped to handle emotional and psychological changes with the transition to motherhood (Erfina et al., 2019), which increases their vulnerability to the psychosocial problems of parenting experiences (Dinwiddie et al., 2018; Parfitt & Ayers, 2014). Teenage mothers often face numerous challenges in meeting the needs of their newborn, adjusting to their new maternal role, lacking social support, and experiencing social isolation (Angley et al., 2015). They often feel devalued and face judgement for becoming mothers at an adolescent age and are considered to deviate from social norms, which exposes them to rejection, stigma, and negative reactions from families and peers (Mangeli et al., 2017). These factors can contribute to intense emotional disturbance after childbirth (James et al., 2012).

The mental health disorders that occur following childbirth include postpartum blues, postpartum depression, and postpartum psychosis (Mughal et al., 2022). Postpartum depression (PPD) is a major depressive disorder that occurs within the first 6 weeks of childbirth but may persist through the 12th month of the postnatal period (Roehr, 2013). While symptoms of PPD may vary among mothers, the majority of women experience loss of interest, fatigue, sadness, withdrawal from social engagement, decreased parenting functioning, and in some cases, thoughts of suicide or infanticide (O'hara & McCabe, 2013). The prevalence of PPD among teenage mothers is estimated to be 25%, compared to 10% among adult mothers (Kingston et al., 2012).

Teenage mothers who develop PPD may exhibit poor health-promoting behaviors, and their newborns may experience developmental delays and negative social interactions in their future lives (Kleiber & Dimidjian, 2014). Additionally, PPD affects the parenting function of mothers, and newborns may not be exclusively breastfeeding, exposing them to malnutrition and intestinal problems (Badr et al., 2018; Layton et al., 2021). Although it is impossible to predict who will develop postpartum mental illness, existing research has identified psychosocial problems, including socioeconomic factors, stressful life events, lack of social support, and discrepancies in motherhood realities and expectations as the main risk factors for postpartum depression (Alshikh et al., 2021; Zhong et al., 2018). Psychosocial interventions are nonpharmacological interventions that target psychological and social factors rather than biological predictors (Ganslev et al., 2020), including, but not limited to, group or individual psychotherapies, nondirective counseling and social support (Barbui et al., 2020).

Cognitive behavioral therapy (CBT) is an intervention that aims to address unhelpful thoughts, beliefs, and behaviors that contribute to depression symptoms. On the other hand, psychoeducation interventions focus on providing information to people about their condition and empowering them in the management of their issues (Beck, 2020; Department of Health, 2001). Interpersonal therapy is a nonjudgmental intervention that helps people address current challenges and improve mental health by building supportive social networks and developing effective communication skills (Weissman & Markowitz, 2002). Social support interventions are derived from social support theory, which encompasses various types of support. These include emotional support, which provides an empathetic and understanding response to mothers’ feelings; information support, which delivers important health information and addresses postpartum challenges for mothers; instrumental support, which provides assistance to mothers in practicing newborn care and training them in how to seek help from others; and appraisal support, which involves listening to mothers’ problems and questions while providing verbal encouragement and admiration (House, 1983).

Although previous reviews have evaluated the impact of psychosocial interventions on preventing PPD among all women, these findings cannot be readily generalized to teenage mothers due to their unique psychosocial concerns during the motherhood transition (Campos et al., 2023; Dennis, 2005; Leis et al., 2009). Furthermore, it is important to note that all studies have been conducted in high-income countries, which presents challenges in extrapolating the findings to low-income countries (Campos et al., 2023; Dennis, 2005; Laurenzi et al., 2020; Leis et al., 2009; Sangsawang et al., 2019). Moreover, previous reviews have not identified the specific domains of psychosocial interventions or the optimal effective time for initiating the interventions (Dinwiddie et al., 2018; Laurenzi et al., 2020; Sangsawang et al., 2019). Therefore, the objective of this review was to investigate the effectiveness of psychosocial interventions for preventing PPD specifically among teenage mothers.

## Methods

The review procedures followed the simplified Preferred Reporting Items for Systematic Review and Meta-Analysis (PRISMA) 2020 guidelines (Page et al., 2021). This systematic review and meta-analysis is not registered with PROSPERO.

### Search Strategy

Five commonly cited online databases, namely, PubMed, Scopus, EMBASE, CINAHL (via EBSCOHost), and the Cochrane Library, were used to retrieve published or unpublished articles between September 20, 2023, to October 30, 2023. The article search was performed using the Population, Intervention, Control, and Outcome (PICO) model (Leonardo, 2018). In addition, a manual reference networking search was conducted (Supplementary file 1). The search terms included “adolescent mothers” OR “teenage mothers” AND “psychosocial interventions” OR “social support interventions” OR “cognitive behavioural therapy” OR “interpersonal therapy” OR “psychoeducation” OR “emotional support” OR “peer support” OR “professional support” OR “information support” AND “postpartum depression” OR “postnatal depression.”

### The Eligibility Criteria

The inclusion criteria were as follows: (1) studies that assessed psychosocial interventions in preventing postpartum depression; (2) the study participants were pregnant or postpartum teenage mothers aged 10–19 years; (3) the studies should be conducted with an RCT study design and published since the initiation of the Millennium Development Goals (MDGs), the eight developmental goals implemented from 2000 to 2015 to improve people’s lives, to include recent articles. Studies including both teenage mothers and adult mothers were eligible for this review, once they had a separate finding for teenage mothers. The control group received the usual antenatal and postnatal services. Articles were excluded if the study participants had already experienced symptoms of postpartum depression at baseline. This implies that the participants’ postpartum depression scores were above the cut-off points indicating depression symptoms, and those participants required therapy rather than preventive interventions. Prevention interventions are more beneficial than treatment interventions for several reasons. First, prevention interventions focus on improving health outcomes before the onset of the disease, providing a better opportunity to avoid complications (Levine et al., 2019). Second, preventive interventions are cost-effective and help alleviate the burden on the healthcare system by decreasing the number of individuals who need treatment for preventable diseases (Meertens et al., 2013). In addition, articles reported in languages other than English, literature reviews, previous reviews with or without meta-analyses, and quasi-experimental studies were excluded.

### Study Selection and Data Extraction

After duplicated articles were removed, the titles and abstracts of each study were reviewed, and when the titles and abstracts did not provide relevant information to evaluate the articles, the full texts of potentially included articles were screened to verify their eligibility. Two authors (LAT and CB) independently conducted the article search and extraction, and any discrepancies were resolved through discussion with another senior author (FWN). Generally, it is useful to specify exactly what interventions are provided, who provided them, how the interventions are delivered, where the interventions are delivered, and when and how many sessions are delivered.

The information extracted included the authors’ name, study setting, publication year, target population, intervention frameworks, detailed intervention activities, intervention initiation period, intervention providers, primary outcome measurement tools, outcome assessment period, and mean PPD scores with standard deviations. During the data extraction, two articles (Barlow et al., 2006, 2013) did not report the standard deviations of each group. Although the corresponding authors were emailed to provide more information about the missing data (i.e., SDs), no further information was obtained. However, based on Cochran’s handbooks for systematic reviews of interventions, the missing standard deviations were calculated using the confidence intervals (CIs) of the mean difference, the sample size of each group, and the *t* value (Higgins & Green, 2008a). After the articles were selected and evaluated by the inclusion and exclusion criteria, the included articles were categorized and organized based on the research questions. Subgroups were considered based on intervention type, time of intervention provision, and outcome measurement period.

### Quality Assessment

The Cochrane Collaboration risk of bias (RoB2) tool was used to assess the quality of the included articles, and the risk of bias graph was used to evaluate the potential sources of bias, including selection bias, allocation concealment, performance bias, measurement bias, attrition bias, and reporting bias (Sterne et al., 2019).

### Data Analysis

The findings of postpartum depression measured between 6 weeks and 12 months of the postnatal period were synthesized. Statistical analyses were performed using the STATA statistical package, version 17. The standardized mean difference (Hedges’s g) was used as a summary statistic in the meta-analysis to assess the effect size, adjusting for the different psychometric measurement scales of depression. A negative value for the standard mean differences indicated the benefit of the interventions. Since the studies included in the meta-analysis were heterogeneous in terms of intervention frameworks, outcome assessment times, and measurement tools; a random effect model was used to pool the effect sizes (Higgins & Green, 2008b).

The heterogeneity of the effect sizes was estimated using the *I*^2^ statistic and Cochran’s *Q* statistic along with the *p*-value. The *I*^2^ statistic ranges from 0 to 40%, implying that the variation in effect size across the studies is likely due to chance rather than a true difference in the study characteristics, 30–60% indicates moderate heterogeneity, 50–90% represents substantial heterogeneity, and 75–100% shows considerable heterogeneity (Deeks et al., 2019). Cochrane handbooks for a systematic review of interventions suggest exercising caution when interpreting the Chi^2^ test, particularly when the number of included studies is small. This is because the Chi^2^ test has low power in such cases, meaning that the non-statistically significant result may not necessarily indicate the absence of heterogeneity. Therefore, in the analysis, a statistical significance level of 0.1 was used instead of the usual level of 0.05 (Higgins & Green, 2008a).

A Galbraith plot was used to detect potential outlier studies that might be the source of heterogeneity. Subgroup analysis was performed based on the intervention frameworks and the period of intervention provision. Publication bias was assessed through funnel plot asymmetry, Egger’s test, and Duval and Tweedie’s trim-and-fill analysis (StataCorp, 2021).

## Results

### Search Results

**Fig. 1:**
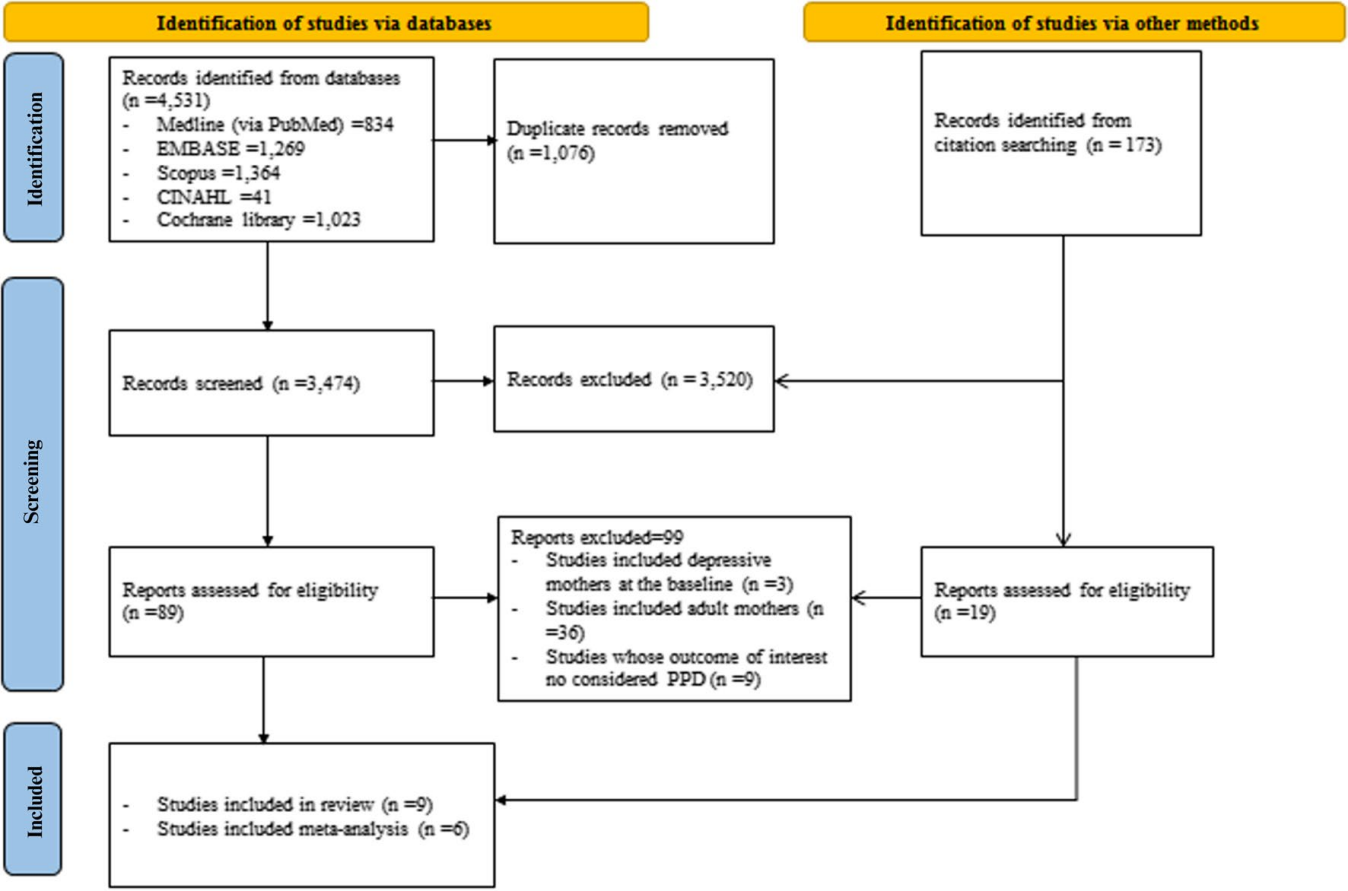
PRISMA flow diagram 2020

A total of 4704 records were retrieved from five online databases and networking searches. Titles and abstracts were used to screen the studies to determine whether they matched the research questions/outcomes of interest based on the inclusion criteria. Full-text article was reviewed to determine eligibility the remaining studies. After removing duplications, 3628 records were assessed by title and abstracts, and then the full texts of 108 articles were screened. Nine articles were included in the review, and six articles were considered in the meta-analysis since the remaining three studies did not report the mean PPD scores with standard deviation and attempts to access these data from the study authors were unsuccessful (Fig. 1).

### Characteristics of the Study

The publication dates of the included studies ranged from 2002 to 2021. Among nine studies, seven were conducted in the United States of America (USA) (Barlow et al., 2006, 2013; Koniak-Griffin et al., 2002; Logsdon et al., 2005; Phipps et al., 2013, 2020; Samankasikorn et al., 2016), one was from Canada (Chyzzy & Dennis, 2019), and one was from Thailand (Sangsawang et al., 2022). The sample sizes of the studies ranged from 33 to 322 participants, and most of the participants were not married. Postpartum depression was the primary outcome in five studies (Chyzzy & Dennis, 2019; Logsdon et al., 2005; Phipps et al., 2013, 2020; Sangsawang et al., 2022). Two studies focused on primiparous teenage mothers (Phipps et al., 2013; Sangsawang et al., 2022). During the baseline assessment, all study participants were examined for depression using assessment tools and found to be below the cut-off points for a diagnosis of depression. Interventions were provided during the antenatal period, the postnatal period, or both (Supplementary file 2).

### Risk of Bias

**Fig. 2:**
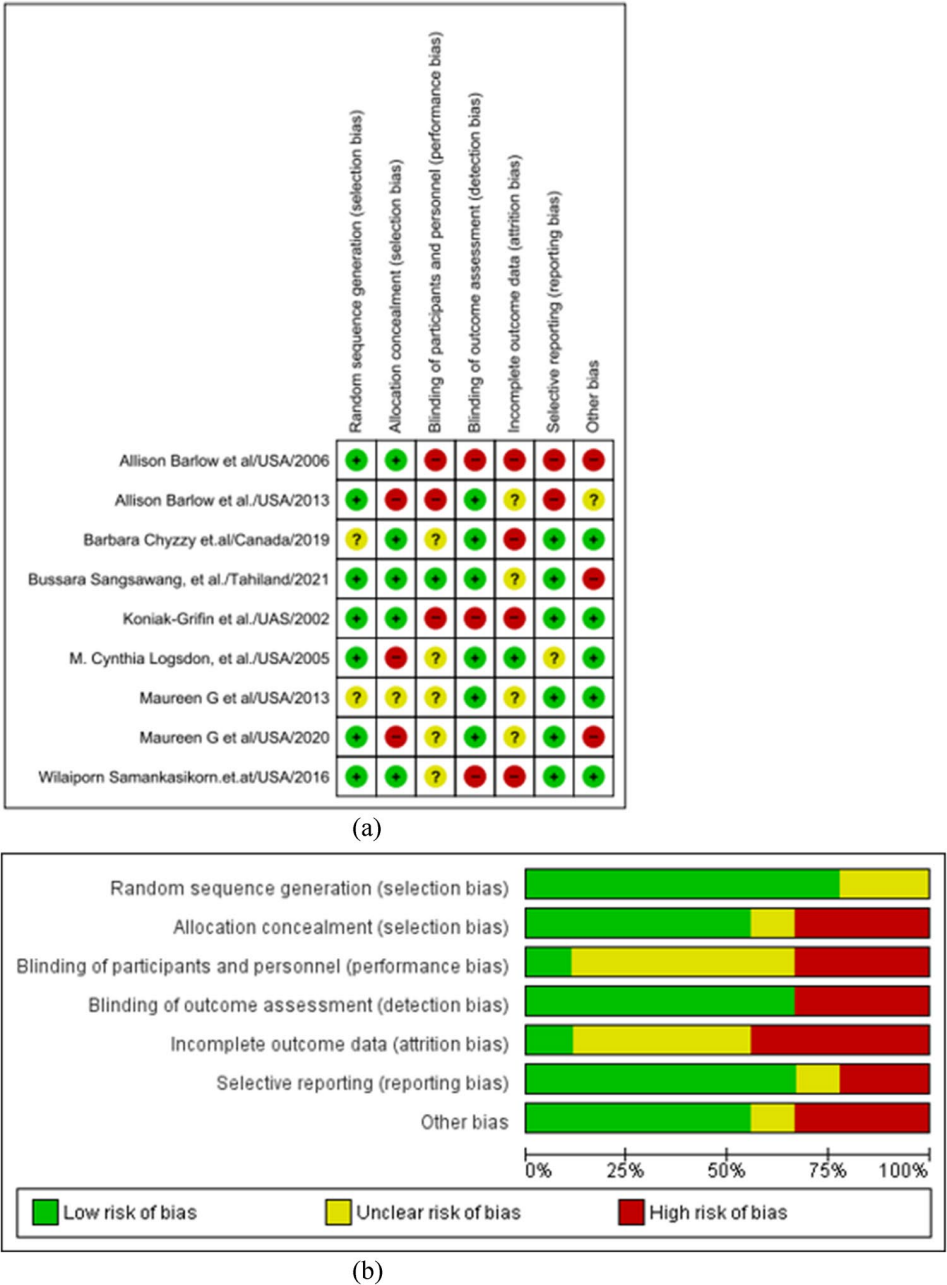
**a** Traffic light plot of risk of bias. **b** Summary plot of risk of bias

The quality of the studies was assessed using the Cochrane risk of bias (RoB2) tool. The included studies showed a range of biases, from a relatively low risk of bias to a high risk of bias (Fig. 2a), and a low risk of bias was observed in the randomization process, while a high risk of bias was reported in terms of participant and research personnel blinding, group allocation concealment, reporting bias, a high attrition rate, and the implementation of appropriate analysis methods (Fig. 2b).

### Intervention Characteristics

The intervention frameworks were social support in six studies (Barlow et al., 2006; Chyzzy & Dennis, 2019; Koniak-Griffin et al., 2002; Logsdon et al., 2005; Samankasikorn et al., 2016; Sangsawang et al., 2022), and psychoeducation in one study (Barlow et al., 2013), while the remaining two studies implemented intrapersonal-based interventions (Phipps et al., 2013, 2020). The content of social support interventions was aimed at preparing teenage mothers for motherhood, raising awareness about the benefit of social support in preventing PPD, and training them on how to seek help from their families or friends. The interventions also included verbal encouragement, providing assistance in postnatal self-care and newborn care, visiting the real-life condition of mothers identifying mothers’ specific needs for assistance, and making referrals for family planning services and mental health services (Chyzzy & Dennis, 2019; Koniak-Griffin et al., 2002; Logsdon et al., 2005; Samankasikorn et al., 2016; Sangsawang et al., 2022).

The psychoeducation intervention aimed to improve women’s motherhood skills and promote psychosocial development. This approach involved the use of real-life scenarios to illustrate various aspects of parenting skills and training participants on how to identify and respond to their baby’s needs (Barlow et al., 2013). Interpersonal interventions included identifying and resolving interpersonal conflicts, improving communication skills, and promoting healthy interpersonal relationships (Phipps et al., 2013, 2020). The intervention activities were included face-to-face contact during the hospitalization period followed by phone calls and weekly home visits lasting for 60–90 min. The interventions included six to forty-three sessions provided starting from the perinatal period up to 1 year of the postpartum period. The interventions were carried out by health professionals, trained paraprofessionals, peers, and primary family members. In most of the studies, the control group received usual antenatal and postnatal care (Supplementary file 2).

### Postpartum Depression Assessment

Four studies used the Centre for Epidemiological Studies Depression Score (CES-D) scale of measurement (Barlow et al., 2006, 2013; Koniak-Griffin et al., 2002; Logsdon et al., 2005). Three studies employed the Edinburgh Postnatal Depression Scale (EPDS) (Chyzzy & Dennis, 2019; Samankasikorn et al., 2016; Sangsawang et al., 2022). The remaining two studies used the Kids and Adolescents Structural Clinical Interview of the DSM-5 (KID-SCID) (Phipps et al., 2013, 2020). Among nine studies included in the review, the three were assessed the outcome of interest at two different time points (Barlow et al., 2006, 2013; Sangsawang et al., 2022). The remaining six studies assessed the outcome of interest at a single time point. The postpartum depression score was measured within the first 3 months of the postpartum period in five studies (Barlow et al., 2006, 2013; Chyzzy & Dennis, 2019; Samankasikorn et al., 2016; Sangsawang et al., 2022), while the measurements were also collected between 6 and 12 months of the postpartum period (Barlow et al., 2006, 2013; Koniak-Griffin et al., 2002; Phipps et al., 2013, 2020) (Supplementary file 2). Due to the difference in the timing of the outcome measurements across the included studies, the overall pooled effect size of the interventions was estimated using the outcome measurement time point (Dennis & Dowswell, 2013).

### Effectiveness of Psychosocial Interventions in Preventing Postpartum Depression

The effectiveness of the interventions in preventing postpartum depression among teenage mothers was determined by comparing the depression score or incidence rate between the intervention group and the control group. Of the nine studies included in this systematic review, only three demonstrated a significant improvement in preventing PPD (Chyzzy & Dennis, 2019; Phipps et al., 2013; Sangsawang et al., 2022). The intervention frameworks that demonstrated a significant benefit in preventing PPD were social support intervention (Chyzzy & Dennis, 2019; Sangsawang et al., 2022) and interpersonal therapy (Phipps et al., 2013). The remaining six studies showed a decrease in the PPD score as a result of psychosocial interventions, but they did not demonstrate a significant difference in the mean PPD score between the intervention and the control groups (Barlow et al., 2006, 2013; Koniak-Griffin et al., 2002; Logsdon et al., 2005; Phipps et al., 2020; Samankasikorn et al., 2016).

### Meta-analysis Results

**Fig. 3:**
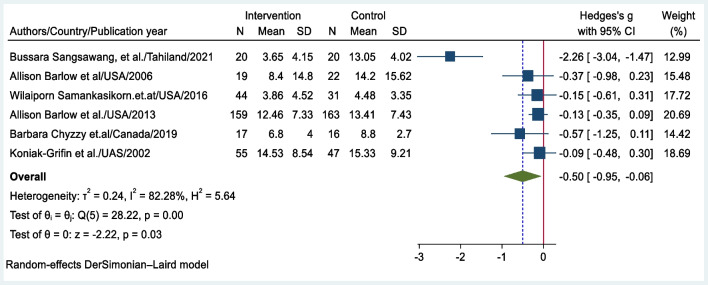
Forest plot of the pooled effect size of psychosocial interventions for preventing postpartum depression among teenage mothers at the last outcome assessment

A meta-analysis was conducted to examine the effect of psychosocial interventions in preventing postpartum depression. The pooled effect size of the six primary studies was − 0.5 (95% CI: − 0.95, − 0.06); thus, psychosocial interventions can significantly decrease the risk of PPD among teenage mothers (Fig. 3).

**Fig. 4:**
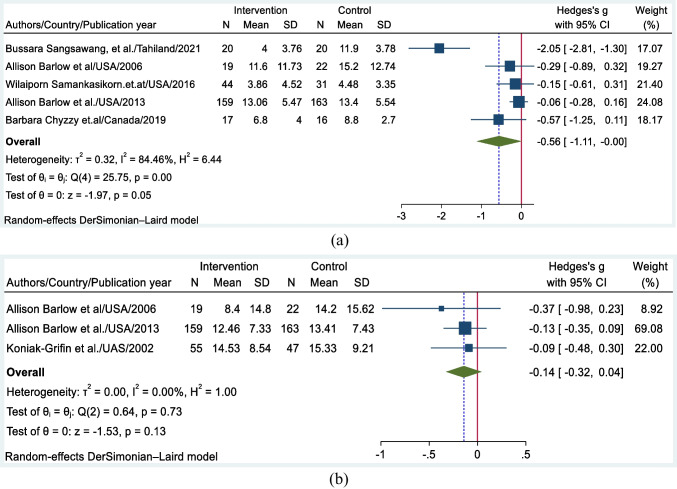
**a** Forest plot of the pooled effect size of psychosocial interventions in preventing postpartum depression measured in the first 3 months of the postpartum period. **b** Forest plot of the pooled effect size of psychosocial interventions for preventing postpartum depression measured between 6 and 12 months postpartum

Pooled effect sizes are also estimated based on the measurement time point of the outcome of interest. Specifically, five studies that measured the PPD score within the first 3 months of the postpartum period, revealed an effect size of − 0.56 (95% CI: − 1.11, − 0.001) (Fig. 4a). Similarly, three studies that measured PPD between 6 and 12 months postpartum yielded an effect size of − 0.14 (95% CI: − 0.32, 0.04) (Fig. 4b). These estimates provide insights into the effectiveness of interventions at different time points during the postpartum period. The larger effect size observed for the measurements within the first 3 months suggests more substantial impacts on reducing PPD symptoms during the early postpartum period.

**Fig. 5:**
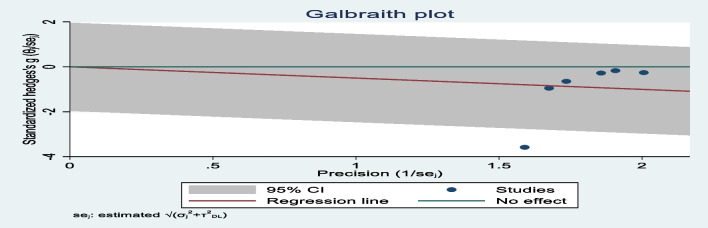
Galbraith plot of heterogeneity in studies of psychosocial interventions for preventing postpartum depression among teenage mothers

Significant heterogeneity was observed between the studies (*p* < 0.001), with a high *I*^2^ = 82.3%. On the Galbraith plot, studies located below the reference line indicated a lower risk of postpartum depression in the intervention group than in the control group. Studies observed above the line of the pooled effect size had a smaller effect size than the overall effect size, while studies presented below the pooled effect size line had a greater effect size. One study had values outside of the 95% CI, suggesting that this study may be a potential source of heterogeneity (Fig. 5).

### Sensitivity Meta-analysis

**Fig. 6:**
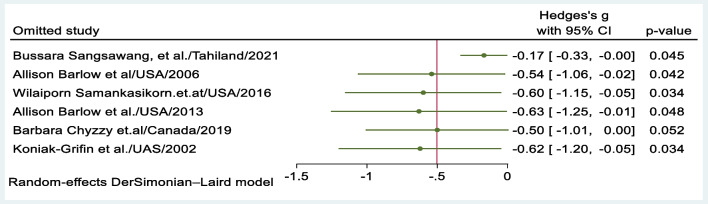
Leave-one-out analysis of study findings of psychosocial interventions for preventing postpartum depression among teenage mothers

The sensitivity analysis showed that when omitting the study conducted in Thailand (Sangsawang et al., 2022), the pooled effect size decreased from − 0.5 to − 0.17, which suggests that the study had a high contribution to the overall effect size. However, the omission of this study increases the precision of the estimate, and a *p*-value less than 0.05 indicates that there was sufficient evidence to conclude that omitting the study had a significant impact on the pooled effect size estimate (Fig. 6). The reason behind this outlier study may be the intervention time, as it was implemented during the postnatal period, and teenage mothers with obstetrical complications such as caesarean delivery or congenital anomalies were excluded from this study.

### Publication Bias

**Fig. 7:**
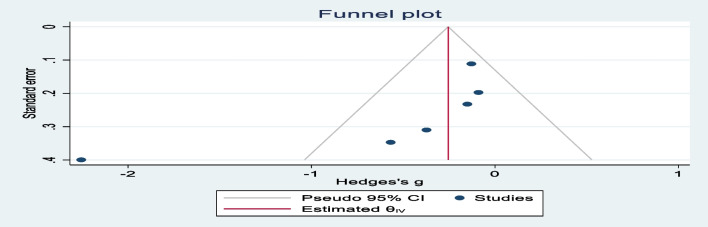
Funnel plot for potential publication bias assessment among studies

The funnel plot appeared to be asymmetrical; studies with larger standard errors were located at the bottom of the plot (Fig. 7). Publication bias was not detected in the trim and fill analyses, and no imputed studies were identified (Table 1). Egger’s test for small-study effects revealed that the estimated slope was − 5.28 with a *p*-value of 0.02, indicating the presence of a small-study effect due to an outlier study.

**Table 1:** Nonparametric trim-and-fill analysis of publication bias

Studies	SMD (Hedges’s g)	95% CI
Observed	− 0.503	− 0.947, − 0.058
Observed + imputed	− 0.503	− 0.947, − 0.058

### Subgroup Analyses

**Fig. 8:**
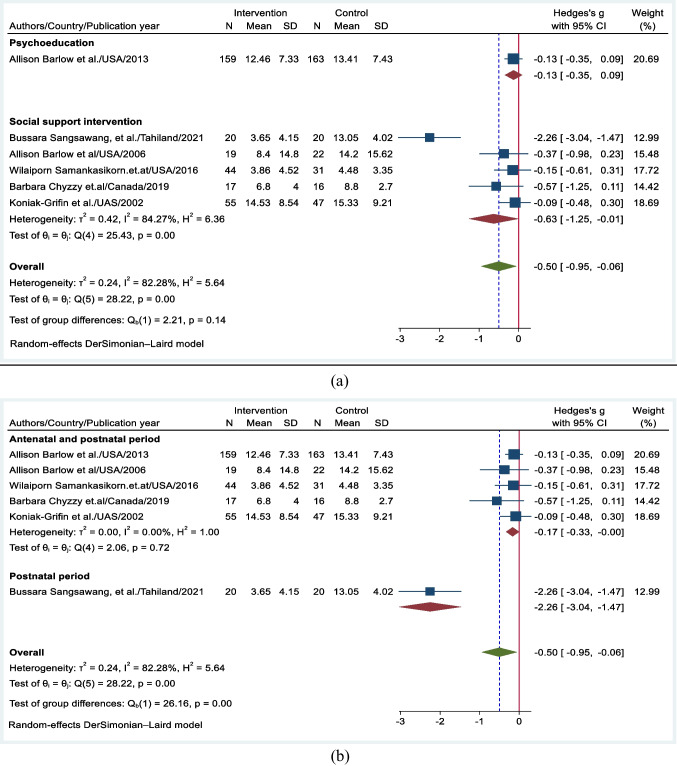
**a** Subgroup analysis based on the types of intervention frameworks. **b** Subgroup analysis based on intervention provision period

Subgroup analyses were conducted based on the type of intervention framework and the time of intervention provision. Social support interventions were more effective at preventing PPD, with an effect size of − 0.63 (95% CI: − 1.25, − 0.01), which was greater than the pooled effect size of − 0.5 (Fig. 8a). Another subgroup analysis based on intervention provision time suggested that interventions initiated in the postnatal period had a greater effect size of − 2.26 (95% CI: − 3.04, − 1.47), while interventions initiated in the antenatal period had a lower effect size of − 0.17 (95% CI: − 0.33, − 0.001) (Fig. 8b).

### Quality of Evidence

To maintain the quality of this study, a comprehensive systematic search strategy was applied to identify the relevant articles, and the quality of each study was evaluated using Cochrane’s risk of bias tool. The extracted data presented the detail intervention constructs, how and where the interventions were delivered, and how many sessions were delivered; and heterogeneity of the studies was assessed by statistical tests. Although this review included randomized controlled trials to ensure the power and quality of evidence, a high risk of bias was observed in the majority of the studies, and the level of heterogeneity between articles was also substantial. Notably, the inclusion of studies with secondary outcomes in the review may have had a potential confounding effect on the findings, which may have contributed to the high heterogeneity observed in the pooled effect size estimation. To decrease this potential effect, subgroup analysis and sensitivity analysis were performed.

## Discussion

This systematic review and meta-analysis included nine primary studies that assessed the effectiveness of psychosocial interventions in preventing postpartum depression among teenage mothers, providing evidence specifically targeting this unique population segment. Overall, while all studies demonstrated a decrease in the risk of developing depression following childbirth, three out of nine studies (Chyzzy & Dennis, 2019; Phipps et al., 2013; Sangsawang et al., 2022) revealed a statistically significant difference between the intervention and control groups. This finding aligns with a previous review that included all mothers and showed that mothers who received psychosocial interventions were significantly less likely to develop PPD than mothers who received usual maternal care alone (Dennis & Dowswell, 2013).

The meta-analysis of six studies showed a pooled effect size of − 0.5 (95% CI: − 0.95, − 0.06), indicating a significant difference in the depression scores between the intervention and control groups. This finding aligns with a previous review among adult mothers, which reported an effect size of − 0.18 between the intervention and control groups (Martin-Gomez et al., 2022). The strength of the effect size discrepancy between these reviews may be attributed to differences in participants’ age; thus, the current review focused on teenage mothers alone, who are more responsive to psychosocial interventions compared to adults (Mohammadi et al., 2016). Similarly, the pooled effect size of the current study is consistent with a previous review that reported an SMD of − 0.68 between the intervention and control groups (Carter et al., 2019). Treatment interventions are more responsive than preventive interventions (Bellon et al., 2015; Conejo-Ceron et al., 2017; Leis et al., 2009; Sockol et al., 2013).

Psychosocial interventions are more effective in preventing postpartum depression in the early postnatal period than in the late postnatal period. The effect size of the intervention in the first three months was − 0.56, but the effectiveness of the interventions decreased after 6 months postpartum, with a small effect size of − 0.14 in the late postnatal period. A previously conducted Cochrane review also demonstrated that psychosocial interventions are more likely to decrease the risk of developing PPD in the early postnatal period. However, the effect of the interventions was not maintained in the late postnatal period, with an effect size of − 0.17 reported after 24 weeks of childbirth (Dennis & Dowswell, 2013). The first 3 months of the postnatal period are a critical time for developing postpartum depression, as women may experience various challenges associated with the new role of parenthood (Dennis, 2005).

Social support and interpersonal interventions showed significant SMDs of depression scores between intervention and control groups. The subgroup analysis suggested that social support intervention was more effective for preventing PPD, with an effect size of − 0.63. Notably, a previous review highlighted that social support interventions have greater benefits in preventing PPD among adult mothers (Campos et al., 2023). Existing evidence also suggests that teenage mothers may be more responsive to social feedback than adult mothers (Letourneau et al., 2004). The effectiveness of the intervention was greater when the social support provision had comprehensive intervention contents, including emotional, informational, and instrumental support (Sangsawang et al., 2022).

Furthermore, the subgroup analysis indicated that interventions implemented exclusively during the postnatal period had a greater effect size, with an effect size of − 2.26. However, due to the presence of an outlier study, caution is needed when interpreting these findings. Psychosocial interventions provided in the postnatal period address the immediate challenges that women encounter after childbirth, allowing these interventions to be successful during a critical period of vulnerability to depression (Stuebe et al., 2018). This finding aligns with previous findings, which revealed that psychosocial interventions provided during the third trimester of pregnancy and/or the early postnatal period are more beneficial for reducing the risk of developing depression symptoms (Dennis, 2005; Dennis & Dowswell, 2013). Another study also demonstrated that psychosocial interventions administered in the postnatal period exhibited a stronger negative association with PPD than did antenatal interventions (Xie et al., 2009).

## Implications

The evidence in this review encourages the integration of simple and inexpensive depression preventive programs in maternal health services to reduce the incidence of postpartum depression (PPD) among teenage mothers. The studies included in this systematic review were conducted in high-income countries alone, which makes it challenging to generalize the findings to low-income countries (LICs). Teenage mothers in low-income countries have many socioeconomic problems, and they may be compelled to endure unplanned pregnancies (Englund & Persson, 2017). In some cases, the financial status of their families forces them to marry wealthy individuals to obtain access to the funds needed for their family well-being (Risenga & Mboweni, 2022). Due to these factors, interventions designed for high-income countries may not be suitable for teenage women residing in LICs, which suggests that researchers should conduct further studies to investigate the impact of psychosocial interventions in preventing postpartum depression among women in LICs to generate conclusive evidence. Although we employed standardized procedures to enhance the power of the evidence, some limitations were observed in this review. First, the selected studies had a high risk of bias, which might have limited the statistical power of the findings and caution is needed when interpreting the findings. This may be justified due to the difficulties of maintaining blinding in preventive interventions since they often lack suitable placebo interventions that mimic preventive measures without providing any benefit, unlike pharmacological interventions. Second, while a greater number of studies is needed to decrease the heterogeneity level of the review, the narrow population segment and the inclusion of only English-language reported articles restricted the number of studies included.

## Conclusion

The evidence from the current systematic review and meta-analysis suggests that psychosocial interventions, particularly social support interventions, effectively decrease the risk of postpartum depression among teenage mothers. The interventions provided during the postnatal period were more beneficial for reducing the risk of developing PPD. Furthermore, the interventions are more beneficial in the first three months of the postpartum period.

## Supplementary Information

Below is the link to the electronic supplementary material.

Supplementary file1 (DOCX 17 kb)

Supplementary file2 (XLSX 23 kb)

## Data Availability

All data analyzed during this review are included in the published article and additional files.

## Abbreviations

*CES-D*: Centre for Epidemiological Studies Depression Score
*CI*: Confidence interval
*KID-SCID*: Kids and Adolescents Structural Clinical Interview of the DSM-5
*PICO*: Population, Intervention, Control, and Outcome
*PPD*: Postpartum depression
*PRISMA*: Preferred Reporting Items for Systematic Review and Meta-analysis
*RCTs*: Randomized control trials
*RoB*: Risk of bias
*SMD*: Standardized mean difference
*USA*: United States of America
