# Supplementary material for: Effectiveness of Psychosocial Interventions in Preventing Postpartum Depression Among Teenage Mothers—Systematic Review and Meta-analysis of Randomized Controlled Trials
Source: Prev Sci. 2024 Oct 3;25(7):1091–103. doi: 10.1007/s11121-024-01728-0 (PMC11519152; doi:10.1007/s11121-024-01728-0)
Supplement: Supplementary file 1 — Supplementary file1 (DOCX 17 kb) [file 11121_2024_1728_MOESM1_ESM.docx]

**Appendix**

**Searching terms**

1. PubMed: ((((((((((((adolescent mothers) OR (teenage mothers)) AND (psychosocial interventions)) OR (social support interventions)) OR (cognitive behavioral therapy)) OR (interpersonal therapy)) OR (psychoeducation)) OR (emotional support)) OR (peer support)) OR (professional support)) OR (information support)) AND (postpartum depression)) OR (postnatal depression)=834
2. EMBASE: ((((((((((((adolescent mothers) OR (teenage mothers)) AND (psychosocial interventions)) OR (social support interventions)) OR (cognitive behavioral therapy)) OR (interpersonal therapy)) OR (psychoeducation)) OR (emotional support)) OR (peer support)) OR (professional support)) OR (information support)) AND (postpartum depression)) OR (postnatal depression)=1269
3. CINHAL(via EbscoHost): ((((((((((((adolescent mothers) OR (teenage mothers)) AND (psychosocial interventions)) OR (social support interventions)) OR (cognitive behavioral therapy)) OR (interpersonal therapy)) OR (psychoeducation)) OR (emotional support)) OR (peer support)) OR (professional support)) OR (information support)) AND (postpartum depression)) OR (postnatal depression)=41
4. Cochrane Library: ((((((((((((adolescent mothers) OR (teenage mothers)) AND (psychosocial interventions)) OR (social support interventions)) OR (cognitive behavioral therapy)) OR (interpersonal therapy)) OR (psychoeducation)) OR (emotional support)) OR (peer support)) OR (professional support)) OR (information support)) AND (postpartum depression)) OR (postnatal depression)=1023
5. Scopus: adolescent mothers OR teenage mothers AND psychosocial interventions OR cognitive behavioral therapy OR psychoeducation OR interpersonal therapy OR social support interventions OR emotional support OR information support OR professional support OR peer support AND postnatal depression OR postpartum depression AND randomized control trial OR RCT =1364
